# The Action of Strophanthus

**Published:** 1888-05

**Authors:** 


The Action of Strophanthus.—Fraenkel sums up his views as follows:

1. Twelve cases of valvular trouble, mostly mitral stenosis and insufficiency, and aortic stenosis. Three cases were complicated by considerable dilatation of the left ventricle, chronic nephritis, interstitial hepatitis and pleuritis dextra. Seven of these did not react to the drug; in four, there was a brilliant success; in one, a moderate one. The maximum dose was twenty to thirty drops in the day. In the improved cases, dyspnœa, œdema, etc., lessened, but the remedy refused to act after some days, because the system had gotten used to it. In the negative cases, digitalis acted excellently four times; moderately well once, and not at all in two.

2. Three cases of arterio-sclerosis, with dilatation and hypertrophy of the left ventricle. Here strophanthus was without effect, while in two of these cases digitalis was of real service. The cardiac asthma was not at all influenced, which is not in accord with Zerner and Löw's nor with Frazer's views.

3. Three cases of functional disorder of the heart—weakened heart. Two from alcohol, one from tobacco. In these cases, with irregular pulse, dropsical effusions and dyspnœa, it acted extraordinarily favorably. One patient received, up to the time of his discharge as cured, in seventeen days, 16.5 grams; another, in twenty-two days, twenty-one grams of the tincture.

4. In chronic nephritis, the results were negative.

5. A case of portal engorgement, with cirrhosis of the liver. The result was a brilliant success, the ascites disappearing.

Fraenkel concludes:

1. Strophanthus exercises a distinctly tonic action on the heart—in proper doses, it may stimulate; it increases blood pressure, increases diuresis, and removes œdema.

2. It acts moderately in valvular affections, kidney troubles, increased arterial pressure, and favorably in functional heart troubles, and possibly in portal congestion.—*Münch Med. Woch.*, No. 3, 1888.

